# Biosynthesis of Silver Nanoparticles Using *Chlamydomonas reinhardtii* and its Inhibitory Effect on Growth and Virulence of *Listeria monocytogenes*

**DOI:** 10.15171/ijb.1310

**Published:** 2017-01-02

**Authors:** Farajollah Shahriari Ahmadi, Abass Tanhaeian, Maziar Habibi Pirkohi

**Affiliations:** Department of Biotechnology and Plant Breeding, College of Agriculture, Ferdowsi University of Mashhad (FUM), Mashhad, Iran

**Keywords:** Biosynthesis, *Chlamydomonas reinhardtii*, Listeriosis

## Abstract

**Background:**

Biosynthesis of nanoparticles using microorganisms, enzymes, and plant extracts is regarded as an alternative to chemical methods. Microalgae appear to be an efficient biological platform for nanoparticle synthesis as they grow rapidly and produce large biomass at lower cost.

**Objectives:**

The possibility of silver nanoparticles biosynthesisby freshwater green microalgae, *Chlamydomonas reinhardtii*, was evaluated. Furthermore, antibacterial properties of the synthesized nanoparticles were investigated via analysis of growth and toxin production of *Listeria monocytogenes*.

**Materials and Methods:**

Silver nanoparticles were synthesized by incubating 47.5 mL of fresh *C. reinhardtii* culture with 2.5 mL of 200 mM AgNO_3_ solution for 48 h. Characterization of the synthesized nano particles was performed by Transmission Electron Microscopy (TEM), Scanning Electron Microscopy (SEM), Energy Dispersive Spectrometry (EDS) and X-ray diffraction analysis (XRD). Concentration of biosynthesized silver nanoparticles was measured by high resolution ICP-OES spectrometer. Inhibitory effect of silver nanoparticles on *L. monocytogenes* growth was measured. Further, the expression of listeriolysin O was investigated by serial microdilution method and Real-Time PCR assay.

**Results:**

Spherical silver nanoparticles with average size of about 10 nm were formed. The particles had inhibitory effects on bacterial growth and antagonist activity on the expression of listeriolysin O.

**Conclusions:**

*C. reinhardtii* has the potential to be used as an effective platform for production of silver and other nanoparticles. Silver nanoparticles had potent antibacterial properties.

## 1. Background

Metal nanoparticles with unique physicochemical‏ properties have attracted much attention during recent‏ years as useful element in electronics, medicine, textile,‏ and sensing among many others (1).‏

Nanoparticles can be synthesized via a variety of‏ techniques; amongst which, chemically synthesized‏ nanoparticles are the most popular approach.‏ Although chemical synthesis seems to be straightforward,‏ the method poses threats to environment and‏ well-beings of organisms (2). In most cases, chemical‏ approaches employ toxic chemicals as reducing‏ agents, organic solvents or non-biodegradable stabilizing‏ agents. Since human are being exposed to noble metal nanoparticles such as gold and silver, development‏ of safe and green processes of synthesis, free‏ from toxic chemicals seems imminent (3).

Alternatives to chemical biosynthesis, being clean‏ and cost effective to produce, have emerged using‏ microorganisms, enzymes, and plant extracts (4). In‏ between, microalgae, unicellular plants, including *C*.‏ *reinhardtii* appear as the most suitable biological platforms‏ for nanoparticle synthesis. The suitability of‏ these organisms is due to their rapid growth and their‏ potential in biomassproductionat lower cost (5).

Silver nanoparticles are mainly used as antimicrobial‏ agents in textiles, wound dressings, and biomedical‏ devices (6). Silver nanoparticles have been frequently‏ tested for antimicrobial effects on a wide range‏ of pathogenic bacteria (7).

The causative agent of listeriosis, *Listeria monocytogenes* is a Gram-positive facultative anaerobic bacterium‏ found in a variety of food products, with devastating‏ effect on human health (8). The pathogenesis of *L. monocytogenes* is mediated by so called virulence factors‏ such as autolysin, positive regulatory factor *prfA*, and the‏ well-known toxin Listeriolysin O. Listeriolysin O is a‏ pore-forming thiol-activated cholesterol-binding cytolysis‏ n, which is encoded by *hly* gene (8).

The present study was conducted to evaluate the‏ biosynthesis of silver nanoparticles by the *C. reinhardtii* and to evaluate the inhibitory effects of the particles‏ on the growth and the expression of Listeriolysin‏ O as the major virulence factor of *L. monocytogenes*.

## 2. Objectives

The main goal of the present study was to evaluate‏ biosynthesis of silver nanoparticles using green‏ microalgae *C. reinhardtii*. Proposing a green costeffective‏ platform for production of nanoparticles is‏ the main philosophy behind the present experiment.‏ Moreover, since silver nanoparticles are believed to be‏ of antimicrobial potential, the effect of the resulting‏ nanoparticles was investigated on a harmful bacterial‏ pathogen that is *L. monocytogenes*.

## 3. Materials and Methods

*3.1. Biosynthesis of Nanoparticles* Healthy culture of *C. reinhardtii* was harvested in‏ logarithmic phase and centrifuged at 2990×g for 15‏ min at 4ºC. After discarding the supernatant, the biomass‏ was washed with sterile water 3×. The biomass‏ was re-suspended in 47.5 mL of distilled water.‏ AgNO_3_ (2.5 mL of the 200 mM) was added to microalgal‏ biomass. Suspension of *C. reinhardtii* free of‏ AgNO_3_ (50 mL) was used as control. In all the experiments,‏ pH was set at 8. The experiment was carried‏ out in triplicate and the cultures were incubated at‏ 25ºC for 48 h.

### 3.2. Characterization

Color change in the culture was observed visually.‏ The reduction of silver ions was monitored by measuring‏ the absorbance of the reaction mixture in a range‏ of wavelength from 300 to 500 nm using UV-Vis spectrophotometer.‏ Morphology of the silver nanoparticles‏ was studied using Transmission Electron Microscopy‏ (TEM). TEM images were taken using Leo 912 AB‏ high resolution transmission electron microscope‏ operating at an accelerating voltage of 120 kV. A sample‏ of the silver nanoparticles solution was placed on‏ the carbon-coated copper grid and dried prior to‏ microscopy. The biosynthesized silver nanoparticles‏ [SNPs] were further studied by Scanning Electron‏ Microscopy (SEM) using LEO 1450VT microscope.‏ For XRD measurement, a sample of silver nanoparticles‏ solutionwas spread in a petri dish and oven dried. The‏ dried sample was taken for XRD analysis using‏ PHILIPS PW1480 X-ray diffractometer. Concentration‏ of 0.2% (w/v) biosynthesized SNPs was measured by‏ high resolution ICP-OES spectrometer (SPECTRO‏ ARCOS, Germany).

### 3.3. Antibacterial Assay

*L. monocytogenes* (RITCC 1624) was purchased‏ from National Centre of Fungi and Bacteria, Iran.‏ Minimum inhibitory concentration (MIC) of the biologically‏ synthesized SNPs was determined using‏ broth microdilution method in a 96-well standard‏ ELISA plate. Luria Bertani (LB) broth containing 105‏ CFU.mL^-1^ of *L. monocytogenes* cells was used as a‏ culture medium. The final concentrations of SNPs‏ were 0, 12.5, 25, 50, 100, 200 and 400 μg.mL^-1^. No‏ organic solvent was used for dissolving SNPs, because‏ such solvents possess their own inhibitory effect on‏ bacteria. The medium was incubated in a shaking incubator‏ at 37ºC for 24 h. The lowest concentration of‏ SNPs inhibiting bacterial growth was assigned as‏ MIC. To monitor growth dynamics of bacterial cells‏ during exposure to SNPs, growth curve at 0, 50 and‏ 400 μg.mL^-1^ of silver nanoparticle was plotted via‏ measuring optical density (600 nm) at different time‏ intervals.

Real-Time PCR assay was conducted to assess‏ expression of Listeriolysin O (*hly*) under SNPs treatment.‏ RNA isolation and cDNA synthesis was conducted‏ following general procedure (9). Primers were‏ 5´-TTTCATCCATGGCACCACC-3´ and 5´-ATCCGCGT‏ GTTTCTTTTCGA-3´. Expression of the virulence gene‏ (*hly*) was quantitatively analyzed using a Real-Time‏ PCR system (Bio Rad). 16S rRNA gene was used as‏ the calibration standard (9). Real-Time PCR was carried‏ out in a 20 μL reaction volume containing 0.5 μM‏ of each primer and 10 μL of SYBR Green Real-time‏ PCR master mix (Genet Bio, South Korea).‏ Quantitative Real-Time PCR experiments were performed‏ in duplicate for each sample.

## 4. Results

**Figure 1:**
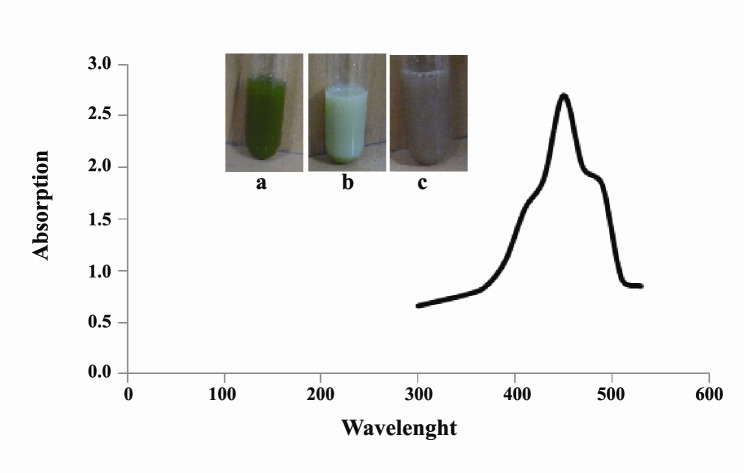
UV-absorption spectrum of *Chlamydomonas reinhardtii* suspension incubated with AgNO_3_. Intersect: color change in *Chlamydomonas reinhardtii* suspension indicating Bioreduction of silver ions into nanoparticles. (a: before addition of silver nitrate, b: immediately after addition of silver nitrate, c: after incubation period)

**Figure 2:**
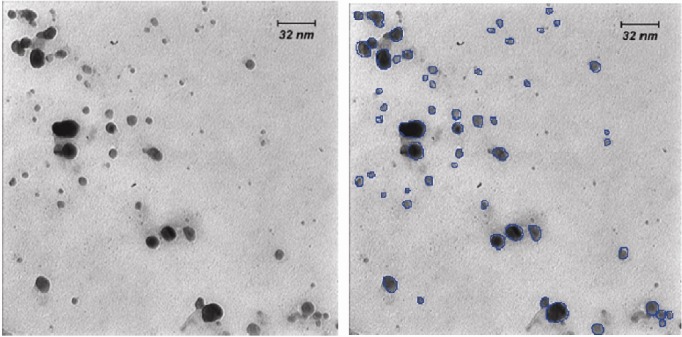
TEM micrograph of biologically synthesized silver nanoparticle (left). Monodispersed particles are highlighted by blue slots (right)

Color change was immediately observed after adding‏ silver nitrate to *C. reinhardtii* suspension. The dark green‏ color of *C. reinhardtii* was changed in to ivory at first‏ and then to brown, indicating biosynthesis of silver‏ nanoparticles. UV-Vis Spectroscopy, a surface plasmon‏ resonance peak was observed at about 450 nm, which‏ confirmed formation of SNPs (Figure 1).‏ Morphology of the biosynthesized SNPs was‏ revealed by TEM microscopy (Figure 2). TEM image‏ was analyzed using X software (4) (Table 1). The silver‏ nanoparticles were about 10 nm spherical with circularity‏ value = 1.

**Table 1:** General properties of the SNPs biosynthesized using *Chlamydomonas reinhardtii*

** Parameter**	** Average value**
Spherical diameter (nm) Width (nm) Length (nm) Roundness Circularity	10.95 6.05 7.38 1.18 1

**Figure 3:**
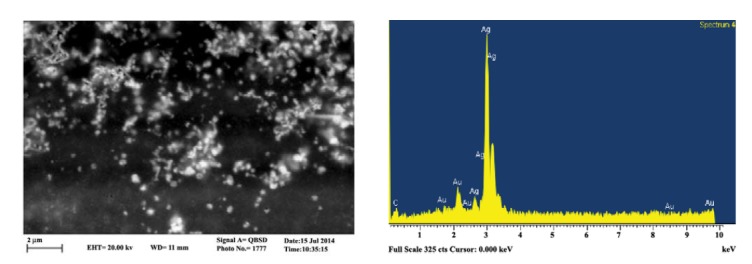
SEM micrograph of silver nanoparticles (left), EDS results indicating sharp peak for silver (Ag^0^, right) (X= energy level, Y=Absorption

‏ Formation of silver nanoparticle was further confirmed‏ by SEM imaging. Energy dispersive spectrometry‏ (EDS) study indicated a sharp signal for Ag, confirming‏ biosynthesis of silver nanoparticles. An‏ absorption peak at 3 keV confirmed presence of silver‏ nanoparticles (Figure 3). The negligible peaks of Au‏ and C in EDS graph weredue to impurities of the solution.

**Figure 4:**
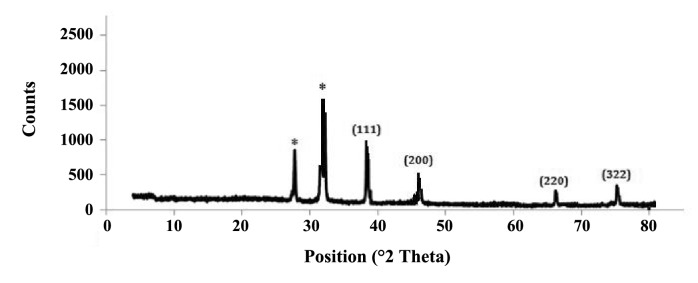
XRD pattern of silver nanoparticles biosynthesized by *Chlamydomonas reinhardtii* biomass

‏ Diffraction properties and crystalline structure of‏ the biosynthesized silver nanoparticles was characterized‏ by X-ray powder diffraction. XRD results showed‏ peaks corresponding to (111), (200), (220) and (322)‏ Bragg reflections (Figure 4). This pattern clearly‏ showed presence of SNPs in the sample. Based on‏ XRD results, particle size can be calculated using‏ Debye-Scherrer formula (2): d=kλ/βcosθ.‏

Where *d* is the size of nanoparticle, *k* stands for‏ Scherrer constant (0.9), λ represents the X-ray wavelength‏ (0.1541 nm), β is the full width at half maximum‏ (FWHM), and θ is diffraction angle.

‏ Using the Scherrer equation the average crystallite‏ sizes of the SNPs was found to be in the range of 9-11‏ nm; confirming particle size estimated by TEM‏ images.‏ ‏ Concentration of the biosynthesized SNPs was determined‏ using ICP method. Results showed that the concentration‏ of SNPs in a 25 mL sample containing both‏ SNPs and *C. reinhardtii* biomass was 2.934 mg.L^-1^ in‏ average.

‏ Serial microdilution was used to evaluate the‏ inhibitory effect of SNPs on growth of *L. monocytogenes*. Results of this test showed that SNPs at the concentration‏ of 50 μg.mL^-1^ can inhibit growth of the‏ pathogen; the minimum inhibitory concentration‏ (MIC) of the SNPs was therefore determined as 50‏ μg.mL^-1^. bacterial suspension showed normal growth‏ below MIC value.

**Figure 5:**
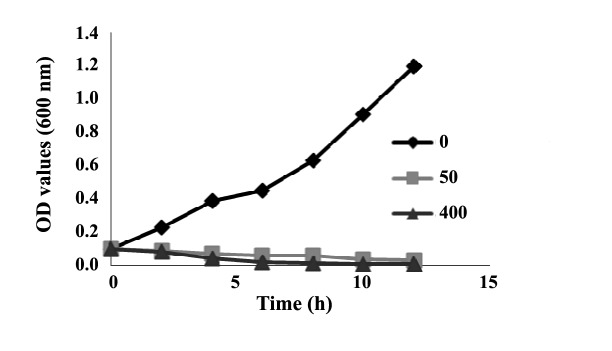
Growth kinetics of *L. monocytogenes* under treatment with different concentrations of biologically synthesized silver nanoparticles

To further study the influence of SNPs on *L. monocytogenes*,‏ growth kinetics of *L. monocytogenes* in a‏ 12 h period was monitored under three concentrations‏ of silver nanoparticles as 0 (control, non-treated), 50‏ μg.mL^-1^ (MIC) and 400 μg.mL^-1^ (the highest concentration‏ in this study). Growth kinetics graph of the bacterium‏ under these SNPs treatments is presented‏ (Figure 5). Control group showed a rapid growth pattern,‏ which reached its maximum level about 12 h after‏ culture. The treated bacterial sample with 50 μg.mL^-1^‏ of SNPs manifested a decreasing growth pattern, so‏ that after 10 h the growth level was nearly zero.‏ Growth decreasing was more severe in the sample‏ treated with 400 μg.mL^-1^ of SNPs, so that a drop of‏ growth was observed only after 4 h, and the bacterial‏ growth was completely inhibited 6 h post treatment.‏

**Figure 6:**
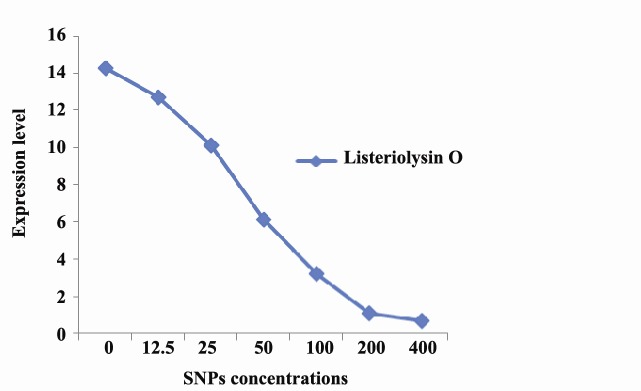
Quantification of listeriolysin O gene (*hly*) expression in various concentrations of SNPs

Influence of SNPs on listeriolysin O -as a virulence‏ factor of *L. monocytogenes* was studied via Real-Time‏ PCR (Figure 6). A dose-dependent decrease in expression‏ of listeriolysin O under treatment with various‏ concentrations of silver nanoparticles was recorded. In‏ the range of 0 to 200 μg.mL^-1^ of SNPs, a nearly linear‏ relation was observed between SNPs concentration‏ and reduction of listeriolysin O expression.

## 5. Discussion

Biosynthesis of metal nanoparticle using a wide‏ range of biological agents offers many advantages‏ over conventional chemical and mechanical synthesis‏ procedures. In the present study, biosynthesis of silver‏ nanoparticles using suspension of *C. reinhardtii* was‏ investigated. Microalgae are natural bio-remediators‏ that due to their surface characteristics accumulate‏ large amount of metal pollutants. During this bioaccumulation‏ procedure, non-toxic metal containing compounds‏ together with nanoparticles are generated from‏ the trapped metal ions (10). *C. reinhardtii* is a green‏ microalga, which naturally occurs in lakes and other‏ fresh water sources (11).

The first indicator of bioreduction of Ag ions to silver‏ nanoparticle is the characteristic change in color of‏ the *C. reinhardtii* suspension. Some theoretical mechanisms have been proposed to explain biosynthesis of‏ nanoparticles by microalgae and *C. reinhardtii* cells.‏ The most probable mechanism is the secretion of cellular‏ reductases into the growth medium by *C. reinhardtii* cells. These enzymes can efficiently reduce silver‏ ions in to silver nanoparticles (12). Moreover,‏ metal ions can be trapped by the carboxylate groups‏ residing on the surface of *C. reinhardtii* cells. The‏ entrapped ions are then reduced by reductase enzymes,‏ which subsequently results in the formation of‏ nanoparticles (13). Presence of a maximum peak at‏ about 450 nm in UV-Vis spectroscopy further confirmed‏ biosynthesis of silver nanoparticles. Surface‏ plasmon resonance peak in the range of 410 nm to 450‏ nm have been reported by other authors as an indicator‏ of SNPs biosynthesis (10,14).

Morphology of the biosynthesized SNPs was analyzed‏ by TEM microscopy. Biologically synthesized‏ nanoparticles can occur in various geometric forms.‏ TEM images of the present study revealed that the‏ biosynthesized silver nanoparticles were spherical‏ with average diameter of 10.95 nm (Table1).‏ Circularity of the biosynthesized SNPs was estimated‏ to be 1, reconfirming spherical shape of the SNPs.‏ Presence of SNPs in *C. reinhardtii* suspension was further‏ confirmed by SEM images, EDS graph and XRD‏ analysis. An absorption peak at 3 keV in EDS study‏ confirmed presence of silver nanoparticles in the solution.‏ The *C. reinhardtii* biomass containing silver‏ nanoparticles was dried and powdered for XRD analysis.‏ Four peaks corresponding to (111), (200), (220)‏ and (322) Bragg reflections were observed in this‏ analysis. The XRD pattern obtained in this study was‏ in accordance with previously determined Bragg‏ reflections associated with silver nanoparticles (15,7).‏ Particle sizeswere estimated using XRD data and‏ Debye-Scherrer formula indicating that the biosynthesized‏ SNPs were 9-11 nm in average, which agrees‏ with TEM microscopy results.

After characterization of SNPs, their antimicrobial‏ effect on *L. monocytogenes* was studied by serial‏ microdilution method. *In vitro* microdilution test‏ showed that SNPs can inhibit the pathogen growth at‏ concentration of 50 μg.mL^-1^. Antimicrobial effects of‏ silver nanoparticles have reported by many authors‏ (16,4,13). Silver nanoparticles produced in this study‏ were about 10 nm, which makes them of ideal size for‏ inhibitory effects on bacterial cells. The size of‏ nanoparticles plays critical role in their efficacy to‏ inhibit microbial growth (17). It has been postulated‏ that nanoparticles with smaller sizes have better‏ antimicrobial effect, because they have larger surface‏ area and higher percentage of interaction than bigger‏ particles (16). Inhibitory effect of silver nanoparticles‏ on bacterial growth can occur in many ways; for example,‏ silver nanoparticles can interfere with sulfur containing‏ biomolecules residing on the bacterial membrane,‏ or they may attack bacterial genome and respiratory‏ chain. These interfering effects ultimately result‏ in bacterial cell death (18). In addition to investigating‏ inhibitory effect of the SNPs on growth of *L. monocytogenes*,‏ we evaluated their influence on expression of listeriolysin‏ O as a major virulence factor of pathogen. Our‏ results showed that SNPs, even at concentrations below‏ MIC value, can reduce expression level of listeriolysin‏ O. Negative effect of the biosynthesized SNPs on‏ expression of virulence factors may offer medical implications‏ in developing new antimicrobial medicines.

## Acknowledgements

The authors wish to acknowledge the Ferdowsi‏ University of Mashhad (FUM), Iran for financial support‏ (Code 31416).

## Funding/Support

The research was funded by department of‏ Biotechnology and Plant Breeding, Faculty of‏ Agriculture, Ferdowsi University of Mashhad.
